# Interpretation of Tonsillectomy Outcome Inventory-14 scores: a prospective matched cohort study

**DOI:** 10.1007/s00405-020-05832-z

**Published:** 2020-02-14

**Authors:** Aleksi Laajala, Timo J. Autio, Pasi Ohtonen, Olli-Pekka Alho, Timo J. Koskenkorva

**Affiliations:** 1 Department of Otorhinolaryngology and Head and Neck Surgery, Oulu University Hospital, P.O. Box 5000, 90014 Oulu, Finland; 2 PEDEGO Research Unit, University of Oulu, Oulu, Finland; 3 Medical Research Center Oulu, Oulu, Finland; 4 Division of Operative Care, Oulu University Hospital, Oulu, Finland

**Keywords:** Tonsillitis, Tonsillectomy, Quality-of-life, TOI-14

## Abstract

**Purpose:**

Knowledge of disease-specific instruments enables the evaluation of health- related quality-of-life (QoL) change associated with chronic and recurrent tonsillitis in adults. The main objective was to explore the interpretation of scores according to the throat-related QoL instrument, Tonsillectomy Outcome Inventory-14 (TOI-14), by determining the typical scores in healthy subjects and patients and define the minimum important change (MIC).

**Methods:**

We performed a prospective matched cohort study in a secondary care area of Oulu University Hospital. The surgical cohort consisted of 42 patients referred to tonsillectomy due to recurrent or chronic tonsillitis. The control cohort consisted of 42 age- and sex-matched healthy controls obtained from the escorts of patients in the same hospital. We translated and validated the Finnish TOI-14 instrument and collected TOI-14 scores at entry and at 6 months and compared results to the anchor question.

**Results:**

At entry, the mean TOI-14 scores were significantly higher in the surgical cohort than in the control cohort [mean (95% confidence interval)] 33.0 (27.0–39.1) vs. 5.0 (3.6–6.4), respectively. At 6 months follow-up, the mean TOI-14 scores had improved markedly after tonsillectomy to the level of the control cohort. In the healthy population, the score was in most cases under 15.0 points. In patients, a score of about 20.0 indicated mild symptoms, 30.0 moderate symptoms and 40.0 or higher intense symptoms. The MIC value was 10.0 points.

**Conclusions:**

These results enable the more accurate interpretation of the scores of the only disease-specific QoL instrument for adult throat-related diseases.

**Electronic supplementary material:**

The online version of this article (10.1007/s00405-020-05832-z) contains supplementary material, which is available to authorized users.

## Introduction

Despite the fact that chronic throat-related diseases are common, disease-specific quality-of-life (QoL) instruments for these illnesses are scarce. For adults with chronic tonsillitis, we have merely the Tonsillitis Outcome Inventory-14 (TOI-14), which was developed and validated in German by Skevas et al. [1]

In addition to the validation studies reported by Skevas et al., Roplekar et al. reported that preoperative TOI-14 scores were high among tonsillectomy candidates and Powell et al. showed similarly raised scores among peritonsillar abscess sufferers [2, 3]. Still, we need more information particularly on interpretation of the TOI-14 scores. According to the recommendations of the International Society for Quality of Life Research (ISOQOL) [4], for a QoL measure to be well accepted it must provide scores that are easily interpreted by patients, clinicians, researchers and policy-makers [5]. One must be able to know what a high or low score represents. Moreover, knowing what comprises a meaningful difference or change in the score [minimum important change, (MIC)] from one group to another (or one time to another) would enhance understanding of the outcome being measured. To achieve this, a comparison to a reference or normative group is important.

To explore the interpretation of the TOI-14 scores, we conducted a prospective age- and sex-matched cohort study with adult patients with chronic or recurrent tonsillitis and healthy controls. The Finnish TOI-14 instrument was first validated according to the recommendations of the ISOQOL and Consensus-based Standards for the Selection of Health Measurement Instruments (COSMIN) initiative [5–7].

## Materials and methods

### Study design

This was a prospective matched cohort study.

### Surgical cohort

We selected participants from consecutive patients referred to the ear, nose and throat outpatient department of Oulu University Hospital for tonsillectomy because of recurrent or chronic tonsillitis from August 2017 to May 2018. The clinical criterion for recurrent tonsillitis was 3 or more episodes of tonsillitis within the previous 12 months. These episodes had to be disabling, prevent normal functioning, be severe enough for the patient to seek medical attention, and be thought to involve the palatine tonsils. It was not necessary for culture or antigen tests to have shown infection with group A streptococcus. The diagnostic criteria for chronic tonsillitis were symptoms of chronic sore throat, halitosis, troublesome tonsil stones, and persistently tender cervical nodes together with abnormal clinical findings in the palatine tonsils (chronically infected, scarred tonsils, and tonsil stones). These symptoms had to continue for at least 3 months and be severe enough for the patient to seek for medical care. Our exclusion criteria were age less than 15 years, long-term antibiotic treatment for another disease, chronic disease and pregnancy.

### Control cohort

The control population was obtained during October 2017 and May 2018 from the escorts of patients receiving care in the outpatient ear, nose and throat department at Oulu University Hospital. For each surgical case, one age- (± 5 years) and sex-matched control case was selected and invited to participate in the study.

### Surgical intervention

The surgical cohort underwent total extracapsular removal of both palatine tonsils under general anesthesia. Ear, nose, and throat specialists or residents performed these procedures with either cold dissection or monopolar electrocautery. The patients were discharged the same day.

### Background information, Tonsillectomy Outcome Inventory-14, and anchor question

At entry, we gathered background information, including an email address, from both cohorts with a questionnaire. We collected information about age, tobacco use, allergies, chronic diseases, risk factors for tonsillitis, tonsillitis history, and throat symptoms.

The TOI-14 questionnaire was originally developed and validated in the German language for adults with chronic tonsillitis. This disease-specific QoL instrument comprises 14 questions, which assess the effect of various aspects of throat-related illnesses on patients’ lives. The questions are divided into four subscales: throat-related problems, overall health, resources and psychosocial restrictions. The questions particularly concern the past 6 months of the patients’ lives. The patient answers each question using Likert scales (0 = no problem to 5 = most severe problem). The sum score is formed by adding up the answers, dividing this sum by 70 and multiplying this by 100 to give an adjusted score out of 100 (maximum). The higher the score, the poorer the throat-related QoL.

We translated the questionnaire into Finnish with forward and back translation as suggested by Wild et al. [8]. Briefly, two native Finnish-speaking professional translators did the forward translation from German to Finnish separately. The two versions were critically reviewed and combined as the primary translated version. Next, a native German professional translator performed a back translation. An evaluation group then compared the original and forward/backward translated versions for any critical differences. The translated version was then tested with nine patients who suffered from recurrent or chronic tonsillitis. After reviewing the patients’ comments, a final version of the translation was approved. The English edition of the TOI-14 instrument as presented by Roplekar et al. [2] is presented in the Online Resource.

We also collected information on the overall disturbance suffered by surgical patients from their throat symptoms using a 7-point global rating. This specific anchor question was: How much do the throat symptoms disturb you in your everyday live? The answer choices were 1—not at all, 2—very little, 3—little, 4—moderately, 5—rather much, 6—much, and 7—very much.

The TOI-14 questionnaires and anchor questions were converted into electronic form and sent to the participants via a third-party service [9] at entry and at the 6-month follow-up. Missing responses were enquired after with an email reminder and by phone.

### Statistical methods

For descriptive data, we calculated the mean and standard deviation (SD). To analyse the distributions of the demographic and background characteristics between the matched surgical and control cohorts, McNemar’s test was applied to categorical variables and a paired samples *t* test to continuous variables. A paired samples *t* test was used for comparison between entry and 6-month values for TOI-14 scores in surgical and control cohorts.

The Finnish version of the TOI-14 instrument was validated according to the recommendations of ISOQOL and COSMIN initiative [5, 10]. The analyses made are presented in the Online Resource material.

The TOI-14 scores for the healthy controls and patients for various tonsillar symptoms were then compared by calculating the mean and 95% confidence intervals (CI). The meaning of various TOI-14 scores in the surgical cohort was evaluated by linking the TOI-14 scores with an external criterion (anchor question).

To estimate the minimal important change (MIC) among the surgical cases, a change score was formulated by subtracting the follow-up TOI-14 scores from the baseline scores, then the following suggested distribution-based methods were performed: value of 0.5 SD from the score difference between entry and 6 months postoperatively, standard error of measurement (SEM) [11], and the change in scores corresponding to the small effect size (0.2) [12]. Finally, anchor-based analysis was done. Using the external anchor question, the score difference between the two adjacent levels on a global rating: “not at all impaired” patients and “very mildly impaired patients” was determined. The information from the anchor-based approach was regarded as primary and that from the distribution-based approach as supportive.

## Results

### Participants and enrolment

**Fig. 1:**
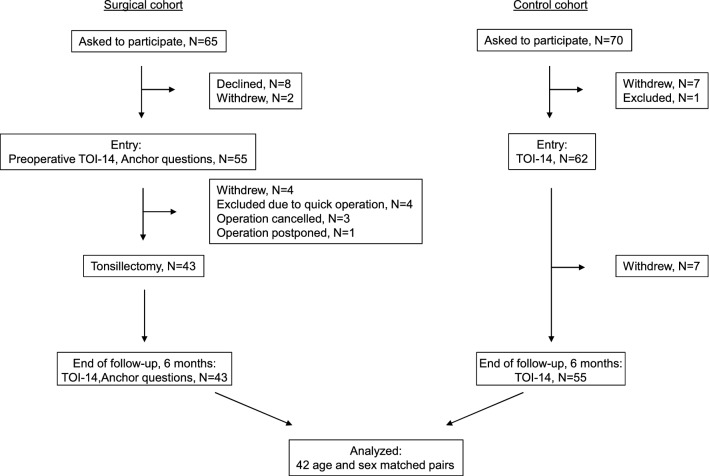
Study profile and participant flow in a matched cohort study with a surgical cohort of 42 patients undergoing tonsillectomy and a control cohort of 42 age- and sex-matched healthy control subjects

*Surgical cohort* A total of 65 candidates underwent screening (Fig. 1). Ten patients declined or withdrew leaving 55 patients who entered the study. Of these, 43 were operated on and answered the questionnaires at 6 months. One further patient had to be excluded due to lack of an appropriate control case leaving 42 patients.

*Control cohort* The total number of invitations sent was 70. The number of controls who consented to participate was 62. Of these, 55 answered the questionnaire at the 6-month follow-up. Of these 55 subjects, we chose 42 age- and sex-matched controls for the surgical cases.

The main indication for the surgery was chronic tonsillitis in 26 cases (62%) and recurrent tonsillitis in 16 (38%) (Table 1). The demographic and baseline characteristics were similar between the cohorts, except that the tonsillar symptoms and upper respiratory tract infections were significantly more common in the surgical cohort (Table 1).

**Table 1:** Demographic and baseline characteristics in age- and sex-matched surgical cohort and control cohort

	Surgical cohort	Control cohort	*p* value^*^
Characteristic			
Number	42	42	–
Age, year, mean (SD)	30 (11)	30 (10)	–
Female sex	36 (86)	36 (86)	–
Tobacco use	5 (12)	3 (7)	0.73
History of allergy	12 (29)	11 (26)	> 0.9
Chronic illness	12 (29)	12 (29)	> 0.9
Risk factors for tonsillitis			
More than four people in the family	14 (33)	16 (38)	0.79
Similar infections in the family	11 (26)	7 (17)	0.42
No. of respiratory infections per year, mean (SD)	3.6 (2.6)	1.0 (1.6)	< 0.001
Untreated dental caries	9 (21)	6 (14)	0.61
Symptoms of gingivitis	7 (17)	6 (14)	> 0.9
No. of toothbrush used per year, mean (SD)	6.8 (3.4)	4.3 (2.0)	< 0.001
No. of prior tonsillitis episodes, mean (SD)			
During the past 6 months	1.1 (1.0)	0	< 0.001
During the past 12 months	2.0 (2.0)	0 (0.3)	< 0.001
Frequent throat pain	36 (86)	5 (12)	< 0.001
Prior complications of tonsillitis			
Peritonsillar abscess	2 (5)	1 (2)	> 0.9
Joint symptoms	3 (7)	2 (5)	> 0.9
Main indication for tonsillectomy			
Recurrent tonsillitis	16 (38)	–	–
Chronic tonsillitis	26 (62)	–	–
Post-tonsillectomy haemorrhage	3 (7)	–	–

Numbers of subjects and percentages in parenthesis unless otherwise stated

*SD* standard deviation

^*^Paired sample *t* test for continuous variables and McNemar test for categorial variables

### Validation of Finnish TOI-14 instrument

According to standards for QoL questionnaire set out by ISOQOL, the Finnish TOI-14 had good psychometric properties. The conceptual and measurement model was meaningful, and the analyses shown in the Online Resource material showed that the instrument showed good reliability, content and construct validity, and responsiveness.

### TOI-14 scores in surgical cohort and control cohort

At entry, the mean TOI-14 scores were significantly higher in the surgical cohort than in the control cohort [mean (95% CI) 33.0 (27.0–39.1) vs. 5.0 (3.6–6.4), respectively]. At the 6-month follow-up, the mean TOI-14 scores had improved markedly after tonsillectomy, whereas those of the control cohort had remained the same [7.1 (3.8–10.4) vs. 5.7 (3.4–8.2), respectively]. The subscales most affected in the surgical cohort were throat-related problems, resources and psychosocial restrictions (Table 2). After tonsillectomy, all these subscales improved, and the scores were similar to those of the control cohort.

**Table 2:** Mean (SD) overall and subscale Tonsillectomy Outcome Inventory-14 scores at entry and at 6 months follow-up

	Entry	Six months	Change (95% CI)
Surgical cohort (*n* = 42)			
Overall score	33.0 (19.3)	7.0 (10.6)	26.0 (19.6 to 32.4)
Throat-related problems	13.0 (5.3)	4.1 (4.6)	8.9 (7.0 to 10.7)
Overall health	5.6 (3.7)	1.6 (3.1)	4.0 (2.9 to 5.1)
Resources and costs	7.3 (7.4)	0.4 (1.1)	6.9 (4.6 to 9.1)
Psychosocial restrictions	7.1 (7.1)	0.9 (3.0)	6.2 (3.9 to 8.4)
Control cohort (*n* = 42)			
Overall score	5.0 (4.6)	5.7 (7.7)	− 0.7 (− 3.1 to 1.6)
Throat-related problems	2.9 (2.4)	3.0 (2.4)	− 0.1 (− 1.3 to 0.9)
Overall health	2.0 (2.3)	1.9 (2.3)	0.1 (− 0.7 to 0.9)
Resources and costs	0.1 (0.7)	0.4 (1.9)	− 0.3 (− 0.9 to 0.3)
Psychosocial restrictions	0 (0)	0.4 (1.7)	− 0.4 (− 0.9 to 0.1)

*SD* standard deviation, *CI* confidence interval

**Fig. 2:**
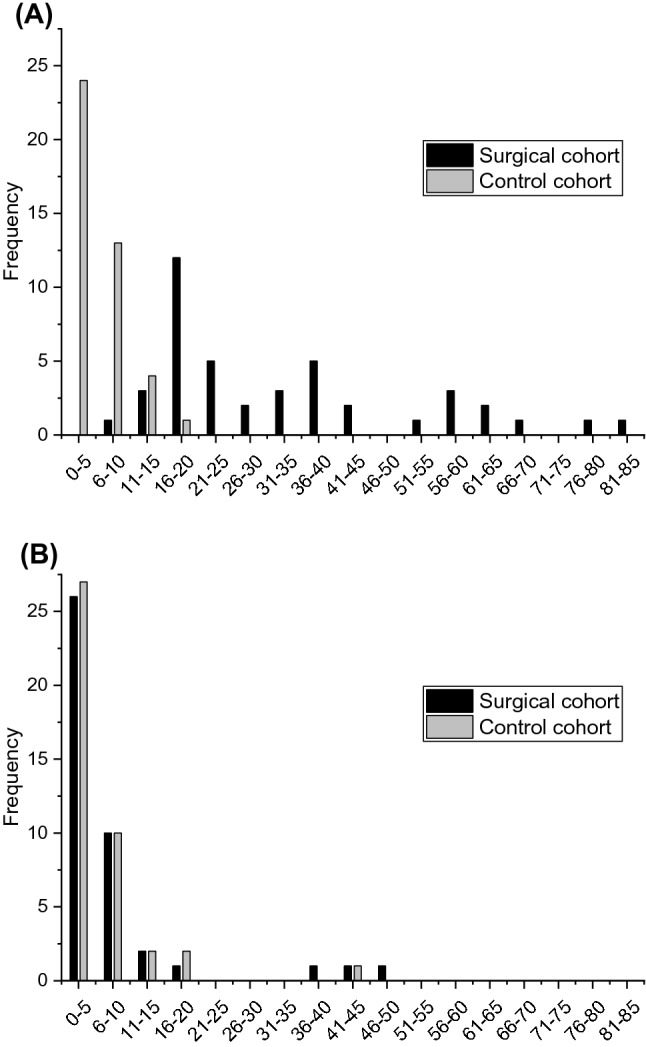
Distribution of Tonsillectomy Outcome Inventory -14 scores **a** at entry, and **b** after the 6-month follow-up in a surgical cohort of 42 patients undergoing tonsillectomy and a control cohort of 42 age- and sex-matched healthy control subjects

At entry, most surgical cases had scores over 15.0 points, whereas most controls had scores of under 15.0 (Fig. 2). After tonsillectomy at the 6-month follow-up, the score distributions in these groups were similar.

Among the surgical cases, those with recurrent tonsillitis episodes had worse mean TOI-14 scores than those with chronic tonsillitis at entry [42.9 (32.1–53.6) vs. 27.0 (20.4–3.7), respectively], but both groups had improved similarly 6 months after tonsillectomy [9.0 (2.5–15.5) vs. 5.9 (2.0–9.7), respectively].

At entry, those surgical cases who thought that their throat symptoms disturbed them very little or only a little had a mean (95% CI) TOI-14 score of 21.4 (11.4–31.5), those who were bothered moderately had 29.6 (22.1–37.1), those with quite much to complain about had 31.8 (21.5–42.1) and those with much or very much had 44.3 (25.4–63.2).

### Determination of minimal important change (MIC): distribution-based methods

In the surgical cohort, the 0.5 SD of the TOI-14 change score was 10.3 points. The SEM value was 3.9 points. Finally, the change in scores corresponding to the small effect size was 3.9 points.

### Determination of minimal important change (MIC): anchor-based methods

The score difference between patients experiencing postoperatively no throat symptoms at all and very mild symptoms on a global rating was 9.9 points. Altogether, 37 (88%) patients had a beneficial change of at least 10 points after tonsillectomy.

## Discussion

We explored TOI-14 scores before and after tonsillectomy in a matched cohort study by collecting information simultaneously from surgical and control cohorts. First, we showed the Finnish TOI-14 instrument to be a valid and reliable instrument in assessing health-related QoL in adults with recurrent and chronic tonsillitis. Second, we found that the adult patients who had recurrent or chronic tonsillitis and who underwent tonsillectomy had substantially impaired QoL defined by the TOI-14 instrument preoperatively compared with the control population. This difference was seen in all subscales, namely throat-related problems, overall health, resources and costs and psychosocial restrictions. Six months after surgery, QoL had improved markedly and the post-operative TOI-14 scores reached the level of the age- and sex-matched control population. The patients with recurrent tonsillitis had higher scores preoperatively than those with chronic tonsillitis, but surgical benefit was seen in both patient groups.

The distribution TOI-14 scores in the control population revealed that, in the healthy population, the score was in most cases under 15.0 points. In patients, the comparison of TOI-14 scores with the external overall rating indicated that the scores of about 20.0 referred to mild symptoms, those of about 30.0 to moderate symptoms and those of 40.0 or higher to intense symptoms. The distribution-based analyses showed that a change in TOI-14 scores of under 4.0 points was likely the result of a measurement error rather than true observed change. The anchor-based analysis demonstrated that the MIC value based on our population was 10.0 points.

The mean TOI-14 score in the present patient cohort for recurrent tonsillitis (42.9, 95% CI 32.1–53.6) was comparable to that reported by Skevas et al. [1] and Roplekar et al. [2] for chronic tonsillitis. In contrast, here, the mean scores for chronic tonsillitis were a bit lower, 27.0 (20.4–33.7). The fact that we may have had looser surgical indications for chronic tonsillitis may explain this difference. Still, 88% of our surgical patients achieved an improvement of at least 10.0 points, which we found to be the MIC value indicating that, despite lower TOI-14 levels at entry, the vast majority of our patients benefitted from tonsillectomy. We found that the mean TOI-14 score in the normal population (5.0, 3.6–6.4) coincides with the findings of both Skevas et al. [1] and Powell et al. [3]

We utilized a matched cohort design, which guaranteed similar methods and timing of data collection and thus reduced the risk of bias when the surgical and control subjects were compared. The TOI-14 instrument was translated into Finnish and psychometrically validated according to the recommendations of the two distinguished societies (ISOQOL and COSMIN). We used electronic methods to collect TOI-14 scores to increase convenience and response rate. As almost all adult Finnish citizens have an email account, a significant selection bias is unlikely. Moreover, the electronic method has been shown to yield similar results to the pencil-and-paper methods in many QoL instruments [13]. We chose an anchor that reflected the patient’s perspective and not that of the clinician, payer or society, as we though the patient’s view was most important when individual treatment is planned. As the baseline figures resemble the figures presented earlier in Finland, we think our results are generalizable to Finland.

The fact that tonsillectomy is among the most common surgical procedure in western societies highlights the need to scientifically evaluate its impact. Well-described disease-specific QoL instruments play a central role in this. Our findings support those of Skevas et al. [1] that TOI-14 as an appropriate tool to evaluate the health-related QoL change associated with chronic tonsillitis but also recurrent tonsillitis in adults.

## Conclusions

In this study, we have explored the TOI-14 instrument in adults and shown the typical scores of both healthy controls and surgical patients with different levels of throat-related symptoms related to recurrent or chronic tonsillitis. Furthermore, we have displayed that an improvement of TOI-14 score of at least 10 points is clinically relevant. This information helps clinicians and researchers to interpret TOI-14 scores in different settings.

## Electronic supplementary material

Below is the link to the electronic supplementary material.

Supplementary file1 (DOCX 39 kb)
