# Supplementary material for: Interpretation of Tonsillectomy Outcome Inventory-14 scores: a prospective matched cohort study
Source: Eur Arch Otorhinolaryngol. 2020 Feb 14;277(5):1499–505. doi: 10.1007/s00405-020-05832-z (PMC7160096; doi:10.1007/s00405-020-05832-z)
Supplement: Supplementary file 1 — Supplementary file1 (DOCX 39 kb) [file 405_2020_5832_MOESM1_ESM.docx]

**Psychometric validation of Finnish TOI-14**

The Tonsillectomy Outcome Inventory-14 (TOI-14) is a validated German patient reported outcome measurement (PROM) instrument for patients with chronic tonsillitis (1). In this online supplementary data, our aim was to translate the German TOI-14 into Finnish and validate the Finnish TOI-14 for patients suffering from chronic or recurrent tonsillitis. English edition of the TOI-14 instrument as presented by Roplekar et al. (2) is presented in Table 1.

**Material and methods**

Enrollment and inclusion and exclusion criteria for the study are explained in the main article. In addition to questionnaires explained in the main article, we collected a generic health-related quality-of-life (HRQoL) questionnaire RAND 36, at entry and at a six-month follow-up. The TOI-14 was repeated two weeks after entry to evaluate test-retest reliability. Questionnaires were sent via a third-party service and answered online (3). There were no missing data, because empty answers were not allowed in the electronic questionnaire. The specific validation analyses are described below.

**Psychometric validation**

Psychometric validation was carried out by the standards recommended by the International Society for Quality of Life Research (ISOQOL) (4). COSMIN taxonomy and criteria for good measurement properties were also used (5).

**1. Conceptual and measurement model**

TOI-14 was developed by Skevas et al. as a disease-specific PROM for patients with chronic tonsillitis.[1] TOI-14 has 14 questions, each scored 0-5 on a Likert scale (No problem (0) – very mild problem (1) – mild or slight problem (2) – moderate problem (3) – severe problem (4) – problem as bad as it could be (5)). Initially the development group defined 28 problems and symptoms in chronic tonsillitis. The original 28 questions were trialed on 33 patients with chronic tonsillitis and, after analysis, the items in the questionnaire were reduced to 14 according to item difficulty, specificity, internal consistency and factor analysis. TOI-14 consists of four subdomains or constructs, which are throat-specific problems (questions 1-4), general health concerns (questions 5-6), resource impact and costs (questions 7-10) and psychosocial impact (questions 11-14).

Our expert group of four ear, nose and throat (ENT) specialists (A.L., T.A., O-P.A., T.K.) found all four subdomains of the Finnish TOI-14 to be important and meaningful aspects of HRQOL concerning chronic tonsillitis and recurrent tonsillitis.

**2. Reliability**

Reliability means the degree of PROM’s accuracy i.e. how much it is free from measurement error. To test the reliability of the Finnish TOI-14, we tested the internal consistency, test-retest reliability and measurement error. To test internal consistency, we used the surgical cohort that had answered the preoperative TOI-14 (n=55). Internal consistency was tested by calculating Cronbach’s alpha in all four subdomains of the questionnaire. Cronbach’s alpha should be between 0.70 and 0.95 (6). For testing test-retest reliability, we used both the surgical and control cohorts (N=107), which answered the TOI-14 questionnaire at entry and two weeks later. The mean (95% Confidence Interval, CI) time between the two tests was 17 (15-19) days. Test-retest reliability was calculated using intraclass correlation coefficients (ICC). ICC should be at least 0.70 (6). Measurement error was tested by defining the standard error of measurement (SEM) for TOI-14 (7).

We found that the internal consistency of the four different subdomains was good. Cronbach’s alpha was high in all the subdomains except for throat-specific problems (Table 2). For throat-specific problems, Crohnbach’s alpha was 0.67, which is a slightly lower figure than the recommended minimum level of reliability. Low internal consistency in throat-specific symptoms may be the result of some throat symptoms being exclusive to each other. Patients suffering from a dry throat (question1) usually do not have thick secretion in the throat (question 2) at the same time. When question 2 was removed from throat-specific symptoms, Crohnbach alpha was 0.73. Still, we found question 2 to be important enough to be kept in the questionnaire. We found ICC to be high for the overall TOI-14 questionnaire and its subscales (Table 3). In our data, SEM was 3.9 points.

**3. Validity**

To examine the validity of the Finnish TOI-14, the degree to which the questionnaire measures the constructs it is supposed to measure, we examined content validity and construct validity. Content validity examines the extent to which the concepts of interest are comprehensively represented by the items in the questionnaire. Construct validity refers to the extent to which scores in a particular instrument relate to other measures in a manner that is consistent with theoretically derived hypotheses concerning the concepts that are being measured. (6)

**3a Content validity**

The TOI-14 questionnaire aims to evaluate the disease burden of chronic tonsillitis and to detect the change in HRQoL after treatment. The recall time for the questionnaire is six months. We consider this to be adequate, so it can capture the chronic aspect of the otherwise common throat symptoms. The concepts the questionnaire is intended to measure are mainly symptoms and functioning.

During the translation process, the Finnish TOI-14 was reviewed by the above-mentioned evaluating group who found the questions to be relevant for both chronic and recurrent tonsillitis. The absence of tonsil stones in throat symptoms in the questionnaire was discussed. Because tonsil stones alone are a relative indication for tonsillectomy, this was not added to the Finnish TOI-14.

During the translation process, the Finnish TOI-14 was tested on nine patients who suffered from chronic or recurrent tonsillitis. Their comments and criticism were reviewed. According to these patients, the Finnish TOI-14 questions were descriptive of their symptoms and easy to interpret.

The characteristics of the participants in the validation study are presented in the main text. Age range was good, 17-61 years of age. Most of the participants were female (36 females, 6 males), but this reflects the normal distribution of the patients referred to tonsillectomy in our hospital.

**3b Construct validity**

To define construct validity, we tested five predefined hypotheses: 1) The TOI-14 scores are elevated in both chronic and recurrent tonsillitis compared to healthy controls 2) the TOI-14 scores are higher in people suffering from recurrent tonsillitis than in those with chronic tonsillitis. 3) Age does not affect the TOI-14 scores. 4) Sex does not affect the TOI-14 scores. 5) The TOI-14 scores are negatively correlated with the RAND36 general health sub-score. Positive or negative correlation coefficients should be at least 0.50. For an accepted construct validity, 75% of the results should be in correspondence with these hypotheses.

We found that, in four out of five hypotheses (80%), the results were as we had anticipated. 1) The mean TOI-14 scores were statistically higher in the surgical cohort that underwent tonsillectomy (n=42) as compared to the control cohort (n=42) (mean (95% CI) 33.0 (27.0 to 39.1) *vs.* 5.0 (3.6 to 6.4).2). The mean TOI-14 scores were higher among patients suffering from recurrent tonsillitis (n=16) than chronic tonsillitis (42.9 (32.1 to 53.6) *vs*. 27.0 (20.4 to 3.7). 3) The mean TOI-14 scores did not associate with age. Participants 30 years of age or older had similar mean preoperative TOI-14 scores to those 29 years of age or lower (mean (95% CI) 29,2 (20.6 to 37.8) *vs.* 35.4 (27.0 to 43.9, respectively). Our hypothesis that there would be no difference between males and females turned out to be wrong. TOI-14 preoperative scores were significantly lower among males (n=6) compared to females (n=36) 19.8 (12.6 to 26.9) *vs*. 35.2 (28.5 to 42.0). 5) At entry, the TOI-14 overall scores correlated significantly with the general health scores in RAND36 among patients undergoing tonsillectomy (n=42) (Spearman’s correlation coefficient -0.63).

**4. Responsiveness**

Responsiveness is defined as the ability of a questionnaire to detect important changes over time (6). To test responsiveness, we tested the TOI-14 overall score in the surgical cohort before tonsillectomy and six months after. We hypothesized, that the TOI-14 scores should decline after tonsillectomy. This was tested using a receiver operating characteristic curve, where PROMs area under the curve (AUC) should be >0.70.

The mean (CI 95%) TOI-14 score was 33.1 (27.0-39.1) before tonsillectomy and 7.1 (3.8-10.4) six months after the tonsillectomy. AUC was 0.94, which is considered an excellent result.

**5. Interpretability**

The interpretability of TOI-14 is examined in the main article. We found that TOI-14 scores are higher among patients suffering from recurrent tonsillitis than chronic tonsillitis. In the healthy population, the TOI-14 score was typically below 15.0. TOI-14 scores about 20.0 indicated mild symptoms, scores about 30.0 moderate symptoms and those of 40.0 or higher intense symptoms. We examined the MIC by distribution- and anchor-based methods and suggest that the MIC value for TOI-14 is 10.0.

**6. Translation and cultural adaptation**

Translation of the TOI-14 questionnaire was done according to the protocol proposed by Wild et al (8). Forward translation was done from German to Finnish by two separate native Finnish-speaking professional translators. The two versions were critically reviewed and combined as the primary translated version. Then a third native German-speaking professional translator performed a back translation from Finnish to German and the translation was compared to the original questionnaire by the evaluating team (senior author T.K, German-speaking supervisor). No major differences were found. Harmonization of the translations was done by four Finnish ENT specialists. After a professional review, cognitive debriefing was done with nine patients who suffered from chronic or recurrent tonsillitis. The patients reviewed and commented on the harmonized translation. According to these patients, the Finnish TOI-14 was understandable and easy to interpret. After reviewing the patients’ comments, the final version of the translation was approved.

**7. Patient burden**

TOI-14 should be easy to use for patients, clinicias and researcher. The length of the questionnaire should not be too long and the language should be easy to read and understandable for the target population. (4)

The TOI-14 is a short questionnaire and does not take too much time to answer. In the surgical cohort (n=55), the median time for answering the first set of questionnaires (TOI-14, RAND36 and anchor questions) was 12 minutes. The median time for answering the TOI-14 questionnaire alone for the second time for the surgical and control cohorts was 1 minute and for 91% of participants, the questionnaire took 5 minutes or less to answer (n=106). During the translation process, the nine patients were interviewed about the TOI-14 questionnaire and no problems with language or terms were recorded.

In TOI-14, the initial scores (0-70) are scaled to 0-100. This may clarify the interpretation of the overall scores, but it may also complicate the use of TOI-14 in clinical practice. This problem can be avoided using electronic questionnaires and automatic scaling.

**Summary**

According to the standards for PROM set out by ISOQOL, the Finnish TOI-14 has good psychometric properties. The Finnish TOI-14 shows good content and construct validity, responsiveness and reliability. The difference between male and female scores needs future research with larger sample sizes. The Finnish TOI-14 can be considered a valid tool to assess HRQoL in patients with recurrent and chronic tonsillitis.

**Table 1. TOI-14, English edition(2).**

| Below you will find list of symptoms that may be caused by or related to throat problems. We would like to know more about these and would appreciate your answering the following questions to the best of your ability. There are no right or wrong answers, and only you can provide us with this information. Thank you for your help. | | | | | | |
| --- | --- | --- | --- | --- | --- | --- |
| Considering how severe the problem is when you experience and how often it happens, please rate each item below on how “bad” it is over the last six months by circling the number that corresponds with how you feel using this scale:  if a certain question is not a problem for you, please circle “0”. Please try not to miss any questions. | No problem | Very mild problem | Mild or slight problem | Moderate problem | Severe problem | Problem as bad as it could be |
| 1. Dry throat | 0 | 1 | 2 | 3 | 4 | 5 |
| 2. Thick secretions (catarrh) in the throat | 0 | 1 | 2 | 3 | 4 | 5 |
| 3. Sore throat | 0 | 1 | 2 | 3 | 4 | 5 |
| 4. Swallowing difficulties | 0 | 1 | 2 | 3 | 4 | 5 |
| 5. Feeling ill | 0 | 1 | 2 | 3 | 4 | 5 |
| 6. Reduced ability to work or to do daily chores | 0 | 1 | 2 | 3 | 4 | 5 |
| 7. Frequency of visits to the doctor | 0 | 1 | 2 | 3 | 4 | 5 |
| 8. Costs of doctor visits | 0 | 1 | 2 | 3 | 4 | 5 |
| 9. Frequency of use of antibiotics | 0 | 1 | 2 | 3 | 4 | 5 |
| 10. Costs of medicines | 0 | 1 | 2 | 3 | 4 | 5 |
| 11. Trouble at work as result of missing working days due to tonsillitis / sore throat | 0 | 1 | 2 | 3 | 4 | 5 |
| 12. Reduced participation in events/activities as a result of tonsillitis / sore throat | 0 | 1 | 2 | 3 | 4 | 5 |
| 13. Fewer gatherings with family / friends as a result of tonsillitis / sore throat | 0 | 1 | 2 | 3 | 4 | 5 |
| 14. Feeling depressed as a result of tonsillitis / a sore throat | 0 | 1 | 2 | 3 | 4 | 5 |

| **Table 2. Internal consistency of subscales** |  |
| --- | --- |
|  | Crohnbach’s alpha (95% CI) |
| Throat-specific problems | 0.67 (0.51 to 0.79) |
| General health impact | 0.83 (0.71 to 0.9) |
| Resource impact and costs | 0.91 (0.86 to 0.94) |
| Psychosocial impact | 0.87 (0.81 to 0.92) |

| **Table 3. Test-retest Reliability** |  |
| --- | --- |
|  | ICC (95% CI) |
| TOI-14 | 0.92 (0.88 to 0.95) |
| Throat-specific problems | 0.95 (0.93 to 0.97) |
| General health impact | 0.91 (0.87 to 0.94) |
| Resource impact and costs | 0.92 (0.88 to 0.95) |
| Psychosocial impact | 0.94 (0.91 to 0.96) |

**References**

1. Skevas T, Klingmann C, Plinkert PK, Baumann I. Development and validation of the Tonsillectomy Outcome Inventory 14. HNO. 2012 September 01;60(9):801-6.

2. Roplekar R, Van M, Hussain SS. Does the quality of life impact of tonsillitis correlate with SIGN guideline compliance? An assessment of range and normality. Clin Otolaryngol. 2016 October 01;41(5):481-6.

3. Webropol survey and reporting tool [Internet]. []. Available from: [www.webropol.com](http://www.webropol.com). Accessed 20^th^ Jan 2020.

4. Reeve BB, Wyrwich KW, Wu AW, Velikova G, Terwee CB, Snyder CF, et al. ISOQOL recommends minimum standards for patient-reported outcome measures used in patient-centered outcomes and comparative effectiveness research. Qual Life Res. 2013 October 01;22(8):1889-905.

5. Prinsen CAC, Mokkink LB, Bouter LM, Alonso J, Patrick DL, de Vet, H C W, et al. COSMIN guideline for systematic reviews of patient-reported outcome measures. Qual Life Res. 2018 May 01;27(5):1147-57.

6. Terwee CB, Bot SD, de Boer MR, van der Windt, D A, Knol DL, Dekker J, et al. Quality criteria were proposed for measurement properties of health status questionnaires. J Clin Epidemiol. 2007 January 01;60(1):34-42.

7. Henrica C. W., de Vet, Caroline B. Terwee, Lidwine B. Mokkink, Dirk L. Knol. Measurement in medicine. Practical guides to biostatistics and epidemiology. 1 edition ed. Cambridge University Press; 2011.

8. Wild D, Grove A, Martin M, Eremenco S, McElroy S, Verjee-Lorenz A, et al. Principles of Good Practice for the Translation and Cultural Adaptation Process for Patient-Reported Outcomes (PRO) Measures: report of the ISPOR Task Force for Translation and Cultural Adaptation. Value Health. 2005 April 01;8(2):94-104.
